# Supplementary material for: WordCommentsAnalyzer: A windows software tool for qualitative research
Source: F1000Res. 2018 Sep 4;7:536. Originally published 2018 May 3. [Version 2] doi: 10.12688/f1000research.14819.2 (PMC6137411; doi:10.12688/f1000research.14819.2)
Supplement: Supplementary file 3 [file f1000research-7-17614-s0002.tgz › b2a79496-4ada-47c4-9682-a99ce867836d.zip!/Eight Iranian Health Journals/Archives of Rehabilitation_15.docx]

© Archives of Rehabilitation. This work is licensed under CC BY-NC 4.0 (https://creativecommons.org/licenses/by-nc/4.0/).

Volume & Issue: دوره 15 - شماره 3

Title: تاثیر کفی فانکشنال و کفش طبی بر تعادل ایستایی در کودکان دچار صافی کف پا انعطاف پذیر

http://rehabilitationj.uswr.ac.ir/article-1-1588-fa.html

هدف: صافی کف پا، یکی از بدشکلی‌های رایج در کودکان به شمار می‌رود که در آن قوس طولی داخلی کاهش یا محو می‌شود و اگر در دوران کودکی درمان نشود، ممکن است در بزرگسالی سبب درد و دیگر مشکلات گردد. کفی و کفش طبی عموماً برای درمان صافی کف پا در کودکان تجویز می‌گردد، اما تأثیر بیومکانیکی این وسایل هنوز واضح نیست. هدف این تحقیق مقایسه دو نوع مداخله ارتزی مرسوم در ایران شامل کفی فانکشنال و کفش طبی بر تعادل ایستایی با اندازه‌گیری جا‌به‌جایی مرکز فشار و سرعت نوسان آن در کودکان دچار صافی کف پای انعطاف‌پذیر است. روش بررسی: این مطالعه نیمه‌تجربی بر روی 30 کودک دچار صافی کف پا و 10 کودک سالم به منظور به‌دست‌آوردن داده‌های تعادل در کودکان نرمال با پوشیدن کفش و کفی طبی انجام شد. ابتدا کودکان بر روی صفحه نیروی دستگاه نروکام به‌صورت یک‌پا می‌ایستادند و سپس این کار را با پای مقابل انجام می‌دادند و میزان جا‌به‌جایی مرکز فشار و سرعت نوسان آن ثبت می‌گردید. آزمون آماری اندازه های تکراری جهت مقایسه مداخله اورتزها در هر فرد .و آزمون تی مستقل جهت مقایسه گروه دچار صافی ف پا با گروه سالم استفاده شد. همچنین جهت آنالیز آماری از نرم افزار اس پی اس نسخه 17 استفاده شد. یافته‌ها: نتایج حاصل نشان داد که در گروه دچار کف پای صاف، کفی فانکشنال به‌همراه کفش معمولی و کفش طبی نسبت به حالت پابرهنه سبب کاهش معناداری در میزان جا‌به‌جایی مرکز فشار می‌شدند (P<0/05) و تفاوت معنی‌داری بین دو گروه کفش طبی و کفی فانکشنال با کفش معمولی وجود نداشت (P=0/085). سرعت نوسان مرکز فشار در حالت پابرهنه بیشتر از حالت‌های دیگر بود، اما در مقایسه با کفش طبی در حالت کفی فانکشنال و کفش معمولی سرعت نوسان کاهش یافت (سطح معناداری در این مطالعه 0/05 در نظر گرفته شد). نتیجه‌گیری: با توجه به نتایج مطالعه، تفاوت معناداری در تعادل بین پوشیدن کفش طبی و کفی فانکشنال به‌همراه کفش معمولی در حالت ایستادن بر روی یک‌پا وجود نداشت و با توجه به کاهش میزان جا‌به‌جایی مرکز فشار با کفی فانکشنال در مقایسه با کفش طبی و نیز عدم‌پذیرش کفش طبی توسط کودکان، به نظر رسد که تجویز کفی فانکشنال به همراه کفش معمولی می تواند تجویز مناسبی برای صافی کف پا کودکان باشد.

Title: بررسی اثربخشی تحریکات وستیبولار بر توجه دیداری کودکان مبتلا به نقص توجه/بیش فعالی

http://rehabilitationj.uswr.ac.ir/article-1-1333-fa.html

هدف: پردازش نامناسب اطلاعات حسی بویژه حس وستیبولار در کودکان مبتلا به نقص توجه/بیش فعالی (ADHD) باعث بروز رفتارهایی همچون بی توجهی و بیش فعالی در آنها میشود. در این تحقیق، تاثیر تحریکات تعادلی بر توجه دیداری کودکان ADHD7 تا 12 ساله با استفاده از ابزار IVA-PLUS-CPT (Integrated Visual and Auditory Continuous Performance Test) ،مورد مطالعه قرار گرفت. هدف تعیین تاثیر تحریکات وستیبولار بر توجه و اختصاصاً توجه بینایی میباشد،تا با معرفی نتایج و نحوه اثربخشی این تحریکات که به شیوه رایج مورد استفاده توسط کاردرمانان، طراحی شده است، بتوانیم مسیر روشنی را در کاربرد تحریکات وستیبولار در کاردرمانی کودکان ADHD نمایان کنیم. روش‌ بررسی: تعداد 30 کودک دارای تشخیص ADHD در دامنه سنی 7 تا 12 سال با غلبه دست راست و بهره هوشی نرمال (آزمون وکسلر) پس از کسب رضایت و به طور تصادفی به دو گروه مداخله و کنترل تقسیم شدند. هردو گروه، بمدت 10 جلسه، بصورت هفته ای دو بار به مدت 5 هفته در فعالیت های معمول کاردرمانی شرکت نمودند با این تفاوت که گروه مداخله تحریکات وستیبولار را نیز در زمان جلسات کاردرمانی طبق پروتکل تعیین شده، دریافت نموند. قبل و بعد از مداخله از تمامی شرکت کنندگان آزمون (IVA –PLUS-CPT) گرفته شد. اطلاعات بدست آمده با استفاده از نرم افزار16 SPSS تجزیه و تحلیل شده و تغییرات پس آزمون و پیش آزمون بین دو گروه نیز با آزمون t مستقل مقایسه شد. یافته‌ها: تحریکات تعادلی منجر به تفاوت معنادار در متغیرهای توجه، توجه دیداری ، شد(P<0/05) ولی در ارتباط با متغیر کنترل تکانه دیداری تفاوت دو گروه معنادار نبود (P>0/05). نتیجه‌گیری: استفاده از تحریکات وستیبولار منجر به افزایش توجه دیداری کودکان ADHD میشود و لذا میتواند به عنوان تکنیک درمانی در درمان این کودکان مورد استفاده قرار گیرد.

Title: نقش حمایت اجتماعی ادراک‌شده در پیش‌بینی اختلالات روانشناختی افراد معلول جسمی حرکتی و مقایسه آن با افراد عادی

http://rehabilitationj.uswr.ac.ir/article-1-1459-fa.html

هدف: این مطالعه با هدف بررسی نقش حمایت اجتماعی ادراک‌شده در پیش‌بینی اختلالات روانشناختی افراد دارای معلولیت جسمی حرکتی و مقایسه آن با افراد عادی انجام شد. روش بررسی: در این مطالعه علی‌مقایسه‌ای، 49 نفر از بین معلولین جسمی حرکتی با روش نمونه‌گیری تصادفی ساده و 50 نفر غیرمعلول از بین افراد عادی با روش نمونه‌گیری در دسترس انتخاب شدند. داده‌ها با استفاده از مقیاس افسردگی، اضطراب و استرس (DASS-21) و مقیاس چندبعدی حمایت اجتماعی ادراک‌شده (MSPSS)، جمع‌آوری و با ابزار آماری خی‌دو، تحلیل واریانس یک‌عاملی و رگرسیون چندمتغیری تحلیل شد. یافته‌ها: نتایج نشان داد که بین دو گروه معلولین جسمی حرکتی و افراد عادی از لحاظ استرس (0/05>P)، اضطراب، افسردگی و حمایت افراد مهم (0/01>P) تفاوت معنی‌دار وجود دارد. همچنین استرس افراد معلول، با حمایت افراد مهم و دوستان؛ افسردگی معلولین با حمایت افراد مهم، دوستان و خانواده و همچنین با نمره کل حمایت اجتماعی؛ و بالاخره اضطراب افراد معلول با حمایت افراد مهم، دوستان و خانواده رابطه معنی‌دار دارد (0/05>P). نتایج تحلیل رگرسیون نیز نشان داد که مدل‌های پیش-بینی استرس، افسردگی و اضطراب از روی حمایت اجتماعی ادراک‌شده در افراد معلول و نیز افراد عادی معنی‌دار است (P<0/01). نتیجه‌گیری: این نتایج تلویحاتی را درمورد پیشگیری از آسیب‌های اجتماعی و روانشناختی به دنبال داشته و در توانبخشی افراد معلول جسمی- حرکتی قابلیت کاربرد دارند.

Title: تأثیر فعالیت های بدنی منتخب بر مهارتهای ادراکی-حرکتی کودکان با ناتوانی های یادگیری عصبی- روان شناختی تحولی پیش از دبستان

http://rehabilitationj.uswr.ac.ir/article-1-1317-fa.html

هدف: یکی از شایع ترین اختلالات در کودکان پیش از دبستان ناتواناییهای عصبی- روان شناختی است. اختلال در انجام حرکات ادراکی – حرکتی از نشانه های این ناتوانی در کودکان است. هدف از پژوهش حاضر تعیین تاثیر فعالیت های بدنی منتخب بر مهارتهای ادراکی- حرکتی کودکان با ناتوانی های یادگیری عصبی- روان شناختی تحولی پیش از دبستان بود. روش بررسی: این مطالعه از نوع تجربی با پیش آزمون و پس آزمون با گروه کنترل می باشد. جامعه ی آماری در این پژوهش کلیه کودکان پیش دبستانی 5 تا 6 ساله مهد کودک ها و مراکز پیش دبستانی شهرستان بابل در سال1392 بود. برای انتخاب نمونه 120نفر از والدین کودکان پرسشنامه عصبی - روان شناختی کانرز را تکمیل کردند و 30 کودک به تصادف (ازطریق نمونه گیری در دسترس) از میان کودکانی که نمره ی بیشتری دراین پرسشنامه دریافت کردند و از شرایط حرکتی مناسب برخودار نبودند بعنوان ملاک ورود به تحقیق انتخاب شدند و به تصادف در دو گروه 15 نفری کنترل و آزمایش قرار گرفتند. برنامۀ حرکتی منتخب در این تحقیق، برنامۀ حرکتی اسپارک بود که شامل فعالیت های تقویتی، بازی و ورزش برای کودکان است که به مدت 15 جلسه(هر جلسه 45 دقیقه و به مدت 35 روز) روی گروه آزمایشی اجرا شد. برای ارزیابی مهارتهای ادراکی- حرکتی کودکان از آزمون حرکتی لینکلن ـ اوزرتسکی استفاده شد. داده ها با روش کوواریانس چندمتغیره مورد تجزیه و تحلیل قرار گرفته اند. یافته‌ها: نتایج به دست آمده نشان داد که برنامه حرکتی منتخب بر بهبود مهارتهای ادراکی-حرکتی کودکان دارای ناتواناییهای یادگیری عصب- روان شناختی موثر بود (P<0/05). تنها در مولفه سرعت حرکت تفاوت معناداری بدست نیامد. نتیجه‌گیری: با توجه به نتایج به دست آمده از این پژوهش می توان نتیجه گرفت که انجام فعالیت های بدنی و ورزشی به عنوان بخش ضروری و اصلی برنامه روزانه این کودکان می تواند نتایج پرثمرتری برای آنها به همراه داشته باشد.

Title: تاتیر 5 هفته تمرینات پروپریوسپتیو بر تعادل پویای بسکتبالیست های مبتلا به اسپرین مزمن مچ پا

http://rehabilitationj.uswr.ac.ir/article-1-1195-fa.html

هدف: بنابه گزارش انجمن ملی ورزش دانشکده ای آمریکا اسپرین مچ پا ازآسیب های رایج دربین ورزشکاران رشته های توپی مختلف از جمله فوتسال و فوتبال و بسکتبال می باشد. که طبق محاسبات انجام شده 10 تا 30%کل آسیب های ورزشی و 80%آسیب های مچ پا را شامل می شود. بررسی تاثیر تمرینات پروپریو سپتیو بر تعادل پویای بسکتبالیست های مبتلا به اسپرین مچ پا. روش بررسی: تحقیق حاضر از نوع نیمه تجربی بود که 30 بسکتبالیست با دامنه سنی 22-18 سال که بطور تصادفی به دو گروه مساوی تجربی وکنترل تقسیم شدندو 5 هفته تمرینات پروپریو سپتیو را انجام دادند . ضمناًتعادل پویای آزمودنی ها قبل و بعد از تمرینات با دستگاه تعادل سنج بایودکس اندازه گیری شد. جهت تجزیه و تحلیل داده ها از نرم افزار spss نسخه 16 استفاده شد. یافته‌ها: نمونه این پژوهش را مردان با میانگین سن (35/1±86/19) گروه تجربی و (23/1 ±66/19) سال گروه کنترل را تشکیل دادند. نتایج آزمون تی نشان داد که در گروه تجربی بعد از اجرای تمرینات پروپریوسپتیو نسبت به قبل از آن در نمره تعادل پویای (عمومی: P=0/001، جانبی: P=0/001 و قدامی-خلفی: P=0/001) تفاوت معنادرای ایجاد شده ولی در گروه کنترل نمره تعادل پویا (عمومی: P=0/550،جانبی: P=0/480، و قدامی- خلفی: P=0/840) تفاوت معنادرای نبوده است.  نتیجه‌گیری: بر اساس نتایج این تحقیق می توان برتوانبخشی صحیح وبه موقع ورزشکاران آسیب دیده از ناحیه مچ پا با تاکید بر تمرینات پروپریوسپتیوبرای جلوگیری از ناتوانی واسپرین مجددو همچنین بهبود پارامتراهای تعادل تاکید کرد.

Title: تأثیر برنامه ایمن سازی روانی بر سبک اسناد بدبینانه دانش آموزان پسر با اختلال نارساخوانی

http://rehabilitationj.uswr.ac.ir/article-1-1498-fa.html

هدف: پژوهش حاضر به منظور تعیین اثر بخشی برنامه ایمن سازی روانی بر سبک اسناد بدبینانه دانش آموزان پسر با اختلال نارساخوانی انجام شد. روش بررسی: در این مطالعه شبه آزمایشی دارای طرح پیش آزمون و پس آزمون با گروه کنترل، از دانش آموزان پسر با اختلال نارساخوانی در مراکز اختلالات یادگیری شهر اصفهان، 30 نفر به روش تصادفی انتخاب شدند و در دو گروه آزمایش و کنترل (هر گروه 15 نفر) قرار گرفتند. بهره هوشی و توانایی خواندن دانش آموزان با استفاده از ماتریس های پیشرونده هوش ریون کودکان و آزمون خواندن به ترتیب اندازه گیری شد. سپس کودکان به پرسشنامه سبک‌های اسناد کودکان (CASQ) قبل و بعد از جلسات آموزشی پاسخ دادند. گروه آزمایش در 10 جلسه هفتگی آموزشی برنامه ایمن سازی روانی (2 جلسه در هفته؛ جلسه‌ای 60 دقیقه) شرکت کردند. یافته‌ها: نتایج تحلیل کوواریانس نشان داد که برنامه ایمن سازی به طور معنادار (P<0/001) موجب کاهش سبک اسناد بدبینانه در گروه آزمایش در مقایسه با گروه کنترل شده است. نتیجه‌گیری: به نظر می رسد کاربرد مداخله‌ ایمن سازی روانی می تواند به عنوان یک برنامه آموزشی و توانبخشی منجر به تغییر در باورهای بدبینانه دانش آموزان پسر دارای اختلال نارساخوانی شود.

Title: بررسی پایایی آزمون ارزیابی عملکردی مهارت های خود مراقبتی (PASS) در بررسی مراقبت از خود بیماران Multiple Sclerosis بزرگسال مراجعه کننده به بخش‌های توانبخشی شهر تهران

http://rehabilitationj.uswr.ac.ir/article-1-1326-fa.html

هدف: فعالیت‌های خود مراقبتی به بخشی از فعالیتها اطلاق می‌شود که فرد به منظور حفظ خویشتن و کسب استقلال در زندگی روزمره‌اش انجام می‌دهد. یکی از آزمون‌های جامع در زمینه ارزیابی فعالیت مراقبت از خود آزمون PASS (Performance Assessment of Self-care Skills) است. هدف از مطالعه حاضر، بررسی پایایی آزمون ارزیابی عملکردی مهارت های خود مراقبتی، در بیماران مبتلا به M.S (Multiple Sclerosis) است. روش بررسی: در این مطالعه جهت بررسی پایایی، آزمون PASS بر روی تعداد 30 نفر از بیماران مبتلا به M.S در شهر تهران طی دو مرحله و با فاصله زمانی دو هفته اجرا شد. مراکز انجام پژوهش عبارت بودند از: مرکز جامع صرع و M.S سینا وابسته به دانشگاه علوم بهزیستی و توانبخشی، مرکز نگهداری معلولین کهریزک : بخش M.S زنان و بخش M.S مردان، انجمن M.S ایران. جهت بررسی تکرارپذیری آزمون PASS از روش آماری ضریب ICC (Intraclass Correlation Coefficient) استفاده شد. تجزیه و تحلیل و توصیف اطلاعات بدست آمده توسط برنامه نرم افزاری SPSS-20 انجام شد. یافته‌ها: پایایی آزمون PASS برای ارزیابی فعالیت های روزمره زندگی بیماران M.S بزرگسال در بعد استقلال 0/9، در بعد ایمنی 0/96 و در نتیجه 0/94 است (P>0/001). پایایی آزمون PASS برای ارزیابی فعالیت های ابزاری زندگی روزمره بیماران M.S بزرگسال در بعد استقلال 0/96، در بعد ایمنی 0/98 و در بعد نتیجه 0/95 است (P<0/001). نتیجه‌گیری: این یافته ها نشان داد که آزمون ارزیابی عملکردی مهارت های خود مراقبتی (PASS)، در هر سه بعد استقلال، ‌ایمنی و نتیجه دارای پایایی بسیار بالایی است و می توان آن را به عنوان ابزاری پایا به منظور بررسی مراقبت از خود بیماران Multiple Sclerosis بزرگسال به کار برد.

Title: بررسی تاثیر آنی کفی دارای حمایت قوس طولی-داخلی و پد متاتارس بر توزیع فشار کف پا در زنان مبتلا به آرتریت روماتوئید

http://rehabilitationj.uswr.ac.ir/article-1-1500-fa.html

هدف: آرتریت روماتوئید یک بیماری التهابی مزمن است که سبب تغییرات ساختاری و عملکردی در مفاصل پا شده و منجر به تغییر توزیع فشار کف پا می گردد. بنابراین هدف از انجام این مطالعه بررسی تاثیر آنی کفی دارای حمایت قوس طولی-داخلی و پد متاتارس بر توزیع فشار کف پایی در زنان مبتلا به آرتریت روماتوئید بوده است. روش بررسی: در این مطالعه شبه تجربی، پانزده داوطلب زن مبتلا به آرتریت روماتوئید با استفاده از روش غیر احتمالی ساده انتخاب شدند. فشار کف پا با استفاده از دستگاه پدار و در چهار منطقه آناتومیکی در دو حالت با کفی و بدون کفی اندازه گیری شد و میانگین حداکثر فشار (کیلوپاسکال) در این نواحی محاسبه گردید. یافته‌ها: کفی دارای حمایت قوس طولی-داخلی و پد متاتارس سبب کاهش معنادار میانگین حداکثر فشار در نواحی پاشنه (P<0/015) و سر متاتارس‌ها (P<0/03) و افزایش معنادار فشار در ناحیه میانی پا (P<0/00) نسبت به حالت بدون کفی شد. در ناحیه انگشتان نیز تفاوت معناداری بین میانگین حداکثر فشار در دو حالت ارزیابی مشاهده نگردید. نتیجه‌گیری: نتایج حاصل از این مطالعه نشان می‌دهد که کفی دارای حمایت قوس طولی-داخلی و پد متاتارس با اصلاح الگوی فشار بر پاشنه و سر متاتارس‌ها در بیماران مبتلا به آرتریت روماتوئید، سبب افزایش فشار در ناحیه میانی پا و کاهش فشار در نواحی پاشنه و سر متاتارس‌ها شده است. در نتیجه این کفی به عنوان یک مداخله درمانی در این بیماری جهت توزیع طبیعی تر فشار کف پا پیشنهاد می‌گردد.
